# Point-of-care ultrasound-guided cannulation versus standard cannulation in haemodialysis vascular access: protocol for a controlled random order crossover pilot and feasibility study

**DOI:** 10.1186/s40814-018-0370-9

**Published:** 2018-11-26

**Authors:** Monica L. Schoch, Judy Currey, Liliana Orellana, Paul N. Bennett, Vicki Smith, Alison M. Hutchinson

**Affiliations:** 1 Deakin University, School of Nursing and Midwifery, Faculty of Health, 1 Gheringhap st, Geelong, Victoria 3220 Australia; 2 Deakin University, Centre for Quality and Patient Safety Research, School of Nursing and Midwifery, Faculty of Health, Geelong, Victoria 3220 Australia; 3 Deakin University, Biostatistic Unit, Faculty of Health, 1 Gheringhap st, Geelong, Victoria 3220 Australia; 4 Medical & Clinical Affairs, Satellite Healthcare, 300 Santana Row, Suite 300, San Jose, CA 95128 USA; 5 Barwon Health Renal Services, 40 Little Fyans st, South Geelong, Victoria, 3220 Australia; 6 Centre for Quality and Patient Safety Research, -Monash Health Partnership, Clayton, Victoria 3168 Australia

**Keywords:** Haemodialysis, Ultrasound, Vascular access, Renal, Point of care, Cannulation, Crossover, Random

## Abstract

**Background:**

Point-of-care ultrasound (POCUS) has been used in various vascular access contexts; however, to date, it has not been widely adopted in haemodialysis clinics. People with end-stage kidney disease receiving haemodialysis require an arteriovenous fistula (AVF), arteriovenous graft (AVG), or central venous access device (CVAD) in order to access their blood for therapy/treatment. Cannulation issues, such as haematoma and extravasation, related to AVFs and AVGs are common. This pilot and feasibility study will assess the feasibility of a randomised controlled trial aimed at evaluating whether POCUS-guided cannulation results in more successful and accurate AVF needle placement than the standard practice of blind cannulation.

**Methods:**

A controlled, random order crossover design will be used to evaluate two clinical conditions: (1) POCUS-guided cannulation and (2) standard practice of blind cannulation, when used by haemodialysis nurses. The feasibility of conducting this type of trial for these two clinical conditions will be assessed through recruitment, retention, and attrition rates; perceptions of acceptability; implementation measures; and assessment of methods of data collection. Clinical outcomes to be assessed are overall cannulation success on first attempt, accuracy of needle tip placement, number of extravasations, procedural time, and patient and nurse perceptions. The setting is a 16-chair dialysis unit in regional Australia. Participants will include adult haemodialysis patients with an AVF in situ for greater than 2 months and haemodialysis-trained registered nurses working full- or part-time. Clinical outcomes will be analysed using generalised linear mixed models. Feasibility data will be reported using descriptive statistics. Qualitative audio data will be digitally recorded, transcribed verbatim, and analysed using thematic analysis.

**Discussion:**

Point-of-care ultrasound for cannulation has the potential to promote high-quality, safe nursing care and decrease cannulation damage for patients on haemodialysis. Due to the lack of evidence for patient benefit and its innovative and niche use in haemodialysis centres, POCUS is currently only specified in one international guideline. This study will inform sample size calculations for a future multi-site trial.

**Trial registration:**

Australian New Zealand Clinical Trials Registry, (21/11/2017) ACTRN12617001569392

## Background

To facilitate the removal of toxins from the blood via haemodialysis, three vascular access options are available: arteriovenous fistula (AVF), arteriovenous graft (AVG), or central venous access device (CVAD). Creation of an AVF involves surgical attachment of a vein to an artery, thus making a hybrid arterialised vein. Although the AVF allows access to the blood, maturation issues can arise from poor arterialisation or vessel calcification, particularly in older patients and in patients with peripheral vascular disease and/or diabetes [1]. Diabetes is currently the leading cause of renal failure among patients starting dialysis [2]. In 1991, approximately 12% of the patients starting on haemodialysis were diagnosed with diabetes compared to 72% in 2016 [3]. In 1987, there was only one reported case (0.2%) of a patient over the age of 75 years starting haemodialysis and only 9% had peripheral vascular disease, compared to 25% and 23%, respectively, in 2016 [3]. Thus, the proportion of patients commencing dialysis with poorer quality vessels is increasing.

An AVF is the patient’s lifeline, and the role of the nurse is to protect and maintain that lifeline with minimal adverse outcomes [4]. Cannulation complications, particularly in relation to constant-site puncture and extravasation are common, leading to poor patient outcomes [5]. An AVF may be deemed clinically ready for cannulation, but the cannulator’s skill level may have an effect on the success or failure of the cannulation process [6]. With rising numbers of patients with poor vessels, the skill level and accuracy of the cannulator are even more critical.

A recent audit of the number of miscannulations experienced by patients in one 16-chair regional satellite haemodialysis unit over a 6-month period showed that between 5 and 25 miscannulations occurred per month. Patients who endure numerous miscannulations, some leading to aborted dialysis sessions, often have a tunnelled, cuffed CVAD inserted [7] to enable dialysis treatment. Central venous access devices are inserted into the internal jugular vein and tunnelled through the superior vena cava, where the tip sits at the junction of the right atrium. Complications with CVADs include malpositioning, fibrin formation blocking the lumen, clots forming inside the lumen, exit site or tunnel infection, and endocarditis, with the latter potentially causing sepsis or death [8]. Use of CVAD in the haemodialysis population, due to their higher susceptibility to infection, is associated with more hospitalisations, higher infection rates, and an increased mortality rate compared with patients who have an AVF or AVG [9]. Given these complications, the ultimate aim of planning and implementing haemodialysis vascular access care is to avoid the use of CVAD whenever possible. Ways to avoid the need for CVAD insertion include increasing the skill level of the cannulators and using point-of-care ultrasound (POCUS) to guide needle insertion to avoid miscannulations, area puncture, and subsequent damage to the AVF or AVG.

Studies in peripheral vein cannulation have shown positive results with the user of POCUS guidance, i.e. improved cannulation accuracy and decreased adverse events. Since 2013, four systematic reviews have been published of randomised controlled trials (RCTs) to test the use of ultrasound-guided peripheral cannulation [10–13]. Conclusions drawn from the systematic reviews were mixed, with two finding evidence (based on cannulation success rates) to support the use of POCUS-guided peripheral cannulation in patients with known difficult access. However, no evidence was found supporting reductions in procedural time or the number of cannulation attempts [10, 13].

Point-of-care ultrasound is currently specified only in the Canadian Association of Nephrology Nurses and Technologists’ (CANNT) recommendations for the management of vascular access. Recommendation 4 (determination of cannulation sites) indicates: ‘The use of portable ultrasound for access assessment and ultrasound-guided cannulations can optimize cannulation and ensure correct needle placement’ ([14], p. 15). However, this claim is only supported by opinion and not by empirical evidence. Purportedly, the lack of uptake of POCUS in current practice is due to the lack of evidence regarding its efficacy in decreasing cannulation adverse events. Therefore, the purpose of this pilot and feasibility study is to assess patient outcomes associated with the use of POCUS and to generate data that will inform a future multi-site study. This study will assess the feasibility of the proposed design and processes and determine the estimated recruitment required to power a larger multi-site study to test the efficacy of the use of POCUS for haemodialysis cannulation.

## Methods/design

### Aims

The overall aims of this project are to (1) examine the feasibility of the study design and acceptability to nurses and patients of the use of POCUS for guided (dynamic) cannulation in haemodialysis patients and (2) promote successful needle insertion for patients undergoing haemodialysis. These overall aims inform specific methodological and clinical aims, respectively, (1) to assess the feasibility of the proposed design and processes and inform sample size calculations for a larger multi-site study to test the efficacy of the use of POCUS for haemodialysis cannulation and (2) to determine whether POCUS-guided needle insertion improves successful cannulation and cannulation accuracy.

For the purposes of this research, successful cannulation is defined as the needle for haemodialysis being inserted without miscannulation or manipulation. Miscannulation is defined as the needle requiring reinsertion/replacement as a result of extravasation or misalignment with the vessel lumen. All POCUS-guided insertions will be performed using dynamic real-time ultrasound guidance.

The feasibility of study design and methods for measurement of POCUS-guided cannulation versus blind cannulation will be assessed using the feasibility framework advanced by Bowen et al. [15]. The following aspects will be evaluated: recruitment, retention, and attrition of both patients and nurses; methods of data collection; acceptability by both patients and nurses; and demand, implementation, and integration (see Table 1).

**Table 1:**
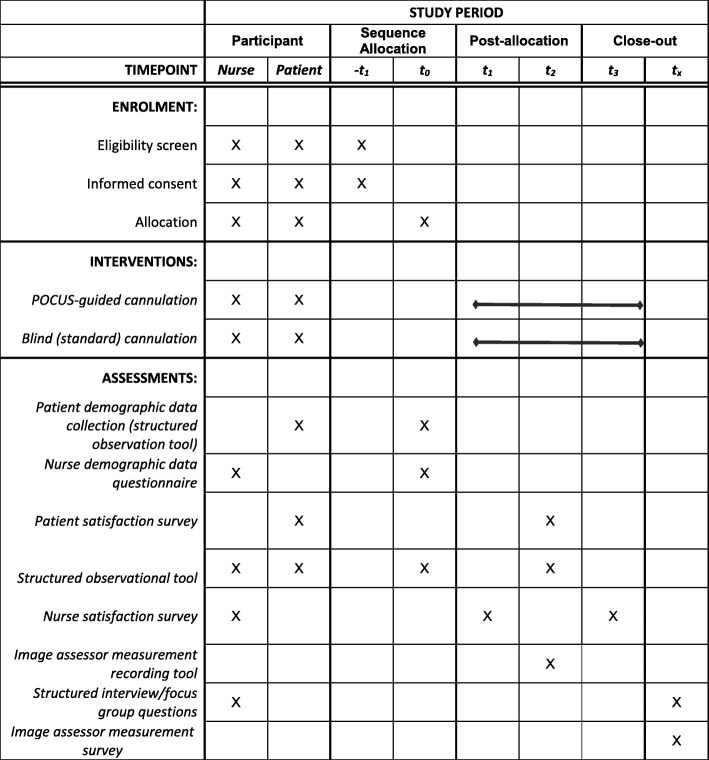
SPIRIT figure illustrating study events and timings.

*-t*_*1*_ prior to allocation; *t*_*0*_ time zero, at allocation; *t*_*1*_ time 1, after the first ultrasound cannulation; *t*_*2*_ time 2, after each cannulation.

To address the first aim (methodological), which is to assess the feasibility of the proposed design and processes and inform sample size calculations for a larger multi-site study to test the efficacy of the use of POCUS guidance for haemodialysis cannulation, the primary outcome measures are:

- Participation rates of patients and nurses (implementation)
- Nurse adherence to and usability of POCUS-guided cannulation in haemodialysis (implementation)
- Procedural time (implementation), patient perceptions of POCUS-guided cannulation (acceptability)
- Nurse perceptions of POCUS-guided cannulation (acceptability, implementation, integration)

To address the second aim (clinical), which is to determine whether POCUS-guided needle insertion improves successful cannulation and cannulation accuracy, the outcome measures assessed at each haemodialysis session are:

- Successful cannulation on first attempt of arterial and venous needles
- Accuracy of arterial and venous needle tip placement
- Total number of extravasations
- Patient pain score

### Design

The study protocol was developed following the SPIRIT (Standard Protocol Items: Recommendations for International Trial) guidelines [16].

A controlled, random order crossover design will be used for this pilot and feasibility study. The study will evaluate two conditions: (1) POCUS-guided cannulation and (2) standard practice of blind cannulation. Each one of up to 20 participant patients will be exposed to each condition implemented by each one of the 10 participant nurses. An independent statistician will produce balanced sequences of conditions and nurses, and a random list to allocate patients to the different sequences at enrolment (each nurse interacting with each patient twice, one using ultrasound and the other using standard blind cannulation). The statistician will be blinded to participant and nurse characteristics. This crossover design (across conditions and nurses) controls for differences between patient characteristics and differences in nurses’ skills, increasing the statistical efficiency, and thus decreasing the overall sample size required for the study [17]. Essentially, each patient within the study acts as his/her own control, thus decreasing the effect of individual factors such as AVF diameter, AVF depth, AVF age, condition of skin, presence of aneurysm or false aneurysm, patient comorbidities, and patient age. In addition, individual nurse factors such as experience with cannulation, ultrasound experience, and self-efficacy will be balanced across all patients.

Quantitative data will be collected at each haemodialysis session to assess whether each cannulation was successful and the location of the needle inside the vessel. At the end of each haemodialysis session, further qualitative data will be collected via structured interviews to elicit patients’ opinions on each cannulation and to identify differences and commonalities in their responses. At the conclusion of the study, nurses will be interviewed using individual or focus groups interviews to collect and analyse their opinions on ultrasound-guided cannulation.

### Setting

The setting for the study is a 16-chair haemodialysis unit in regional Australia. Forty patients receive haemodialysis in the unit each week from Monday to Saturday. The majority of patients undergo haemodialysis three times per week. Twenty registered nurses are employed within the unit on a full- or part-time basis. There are no enrolled nurses or patient care technical staff employed at this site. Data from patients will be collected for 2 days per week (Thursday and Friday) because all potential patients for inclusion attend haemodialysis on these days. Additionally, the patients are expected to be more haemodynamically stable because they have only had 1 day between haemodialysis treatments, as opposed to after the 2-day gap over the weekend when they are more likely to have fluid and electrolyte imbalances. Although a multi-site pilot study would enable an assessment of the feasibility of the study design in different sites, this is an unfunded study that is subject to time constraints. Hence, a pragmatic decision was made to limit the study to a single site.

### Ethical considerations

Ethics approval has been granted from Barwon Health Human Research Ethics Committee (17/112) and Deakin University Research Ethics Committee (2017–367). Registration is approved by the Australian New Zealand Clinical Trials Registry: ACTRN12617001569392 (21/11/2017). Written consent will be obtained from patients and nurses who express interest in participating. Participation is voluntary for all patients and nurses, and they can withdraw at any stage of the study.

### Inclusion and exclusion criteria

*Patients*. The patients included in the study are over the age of 18 years attending the dialysis unit, diagnosed with end-stage renal failure, and receiving haemodialysis three or more times per week via a native AVF will be eligible to participate. The AVF must have been cannulated for routine dialysis treatment for more than 2 months, as the protocol in this dialysis unit is to cannulate all new AVFs with plastic cannulae under either static POCUS assessment or dynamic POCUS guidance for a minimum of 2 weeks and up to 8 weeks, after which patients will change to metal needles (patients new to dialysis start at an acute site within this renal service, not at the study site). Patients must also be using metal needles, as plastic cannulae are generally only used in this unit for new and very difficult cannulations. Patients who cannot give consent or cannot speak English will not be eligible. AVGs are not included in this study as there are no patients with AVG in situ at this dialysis unit and there is only a small percentage used in Australian dialysis units.

*Nurses*. All registered nurses working either full-time or part-time within the unit who have completed the supernumerary haemodialysis training period of 4 weeks will be eligible to participate.

### Target sample size

The target sample size will be 20 patients (based on 30 patients attending the unit with an AVF in situ) and 10 nurses (based on 20 nurses working in the unit). It is estimated that data collection will take place over 12 months. This timeframe is based on a calculation of 10 part-time nursing staff on a rotating roster and takes into account sick and annual leaves and potential patient attrition throughout the study. Nurses will complete both POCUS-guided and blind cannulations on all patients participating in the study in the timeframe.

### Recruitment

All eligible patients and nurses will be invited to participate in the study. The nurse unit manager will give eligible patients a plain language statement detailing the study purpose, procedure, expectations of participants, benefits and risks associated with participation, and the contact details for researchers and Ethic Committee managers. Individuals who express to the nurse unit manager their interest in participating will then be approached by the researcher inperson in the dialysis unit. Information about the study will also be disseminated to eligible nurses in an information session and by email prior to the beginning of the study. Recruitment will continue until the intended sample size is reached.

### Data collection, outcome measures, and procedures

Data collection and management will proceed in accordance with the SPIRIT guidelines as represented in the SPIRIT figure in Table 1.

Baseline demographic data will be collected via a survey (Table 2).

**Table 2:** Baseline data collected at enrolment

Nurses	Patients
Age	Age
Sex	Sex
Dominant hand	Type of AVF
Years of experience in dialysis	Comorbidities
Years of experience with cannulation	Other relevant medical history
Years of experience with ultrasound	Time on haemodialysis
Employment status (full- or part-time)	Frequency of haemodialysis
Completed level of education	Date of AVF creation and first cannulation
Completion of formal or informal ultrasound training	
Frequency of use of ultrasound use to assess AVFs	
Frequency of use of ultrasound to guide cannulation	
Circumstance that prompt to use the ultrasound for guidance	

Nurse participants working at the study site undertake ultrasound competency training on employment and are encouraged to regularly use static POCUS assessment prior to cannulation. The competency training with a vascular access expert involves one training session using ultrasound guidance on training models (namely chicken fillets filled with water balloons).

The aim is for two nurse participants on each morning shift and two nurse participants on each afternoon shift to cannulate two patient participants each during the study. This schedule will capture eight patients per day, 16 patients per week. The patient and nurse participants are each assigned a number (1, 2, 3, etc.) by the researcher based on recruitment order. A statistician, who only knows the total number of patient and nurse participants, will arrange them into dyads via Excel spreadsheet and randomly assign each dyad an allocated condition (POCUS or blind) to be undertaken at first meeting of that dyad (Table 3). At the next meeting of the dyad, the opposite condition would apply (Table 4). Due to the time periods between treatment cannulations and the use of the best practice rope ladder cannulation technique practiced by staff in the unit, evidence of needle placement following use of ultrasound in one session is not anticipated to influence the blind cannulation needle placement in the next session. It is estimated that data collection will take approximately 12 months depending on staff and patient availability.

**Table 3:** Example of patient and nurse allocation to condition

	Nurse 1		Nurse 2		Nurse 3		Nurse 4	
	Dyad	First cond	Dyad	First cond	Dyad	First cond	Dyad	First cond
Patient 1	1	Blind	11	POCUS	21	Blind	31	Blind
Patient 2	2	Blind	12	Blind	22	POCUS	32	POCUS
Patient 3	3	Blind	13	POCUS	23	POCUS	33	POCUS
Patient 4	4	POCUS	14	POCUS	24	Blind	34	Blind
Patient 5	5	Blind	15	Blind	25	POCUS	35	POCUS
Patient 6	6	POCUS	16	POCUS	26	Blind	36	POCUS
Patient 7	7	POCUS	17	POCUS	27	POCUS	37	Blind
Patient 8	8	POCUS	18	Blind	28	Blind	38	Blind
Patient 9	9	POCUS	19	Blind	29	Blind	39	POCUS
Patient 10	10	Blind	20	Blind	30	POCUS	40	Blind

**Table 4:** Example of patient and nurse condition and data collection sheet

		Nurse 1			Nurse 2	
Patient	Dyad	First meeting (condition from randomisation table)	Second meeting (opposite condition)	Dyad	First meeting (condition from randomisation table)	Second meeting (opposite condition)
1	1	Blind	POCUS	11	POCUS	Blind
		20/4/18	6/6/18		4/4/18	11/4/18
2	2	Blind	POCUS	12	Blind	POCUS
		5/10/18	17/12/18		13/7/18	4/8/18

Specific data collected at each haemodialysis session in relation to the nurse cannulator, patient, and dialysis machine are outlined in Table 5. The researcher will observe every cannulation in each treatment session and record the characteristics listed in Table 5 for both the venous and the arterial cannulation. A cannulation will be defined as successful (0) if the needle for haemodialysis is inserted without miscannulation or manipulation, or unsuccessful (1) if the needle extravasates the vessel or blood cannot be accessed from the needle. The number of miscannulations in a session will then take values 0, 1, or 2. For example, a nurse cannulates with the first (arterial) needle and extravasates the vessel, the needle is removed, and pressure applied to the site. This is recorded as ‘1’. In a second attempt, the arterial needle is appropriately placed in the vessel, i.e. it is successful, so this is recorded as ‘0’. The venous needle is also inserted successfully and recorded as ‘0’. The total number of miscannulations for this patient would be ‘1’. In consultation with management of the dialysis unit, the maximum allowable number of blind cannulations for a patient treatment is set at four, a maximum of two cannulations per site. For example, if a nurse miscannulates twice on the arterial point using the standard practice of blind cannulation, they would then use POCUS guidance to cannulate the third time. This information will be recorded by the researcher observing the process. If the cannulator deems POCUS guidance necessary after one miscannulation, this is acceptable and will be documented as above.

**Table 5:** Data collected during the study

Nurses	Patients	Dialysis machine
Sitting or standing to cannulate	Patient arm position	Pump speed (Qb)
Use of gloves (sterile or non-sterile)	Patient-reported pain score (scales 1–10) each needle	Venous and arterial line pressures
Ultrasound probe held longitudinal or transverse	Patient-reported perception of the cannulation experience (scales 1–10)	Any initial pressure alarms
Cannulate in one or two motions	Patient additional comments re-cannulation or ultrasound process	
Needle manipulation once inserted	Ultrasound images in both transverse and longitudinal planes	
Time from tourniquet application until machine started		
Miscannulations (needle removed and re-sited)		
Nurse-reported perceptions of the use of ultrasound

**Table 6:** Feasibility assessments

Tool	Assessing	Participants	Time period
Structured observational tool	Implementation: participation rates (number of participating patients or nurses related to number of eligible patients or nurses). Overall recruitment, retention, and questionnaire completion rates	Patients Nurses	Throughout the data collection phase
Patient satisfaction survey	Acceptability: patient satisfaction with cannulation technique, use of point-of-care ultrasound	Patients	After each cannulation
Nurse satisfaction survey	Acceptability: nurse satisfaction with cannulation technique and use of point-of-care ultrasound	Nurses	After first cannulation with ultrasound After the half way point of the cannulation data collection
Researcher study notes (diary)	Implementation: protocol adherence by nurses; issues verbalised by nurses, other staff, or patients regarding the study protocol, reasons for drop-out for both patients and nurses	Patients and nurses	Throughout data collection phase
Image assessor measurement survey	Implementation: image assessor’s confidence in accuracy of measurements	Image assessor	Following completion of all needle placement measurements
Structured interview or focus group	Acceptability, demand, implementation, and integration: suitability, usability, barriers and facilitators, sustainability	Nurses	Following completion of the cannulation data collection phase

Once haemodialysis is started, the vascular access nurse expert, using the Terason uSmart 3200 T ultrasound, will take static ultrasound images in both transverse and longitudinal planes, save the images to a portable storage device, and print them for image assessor review.

**Fig. 1:**
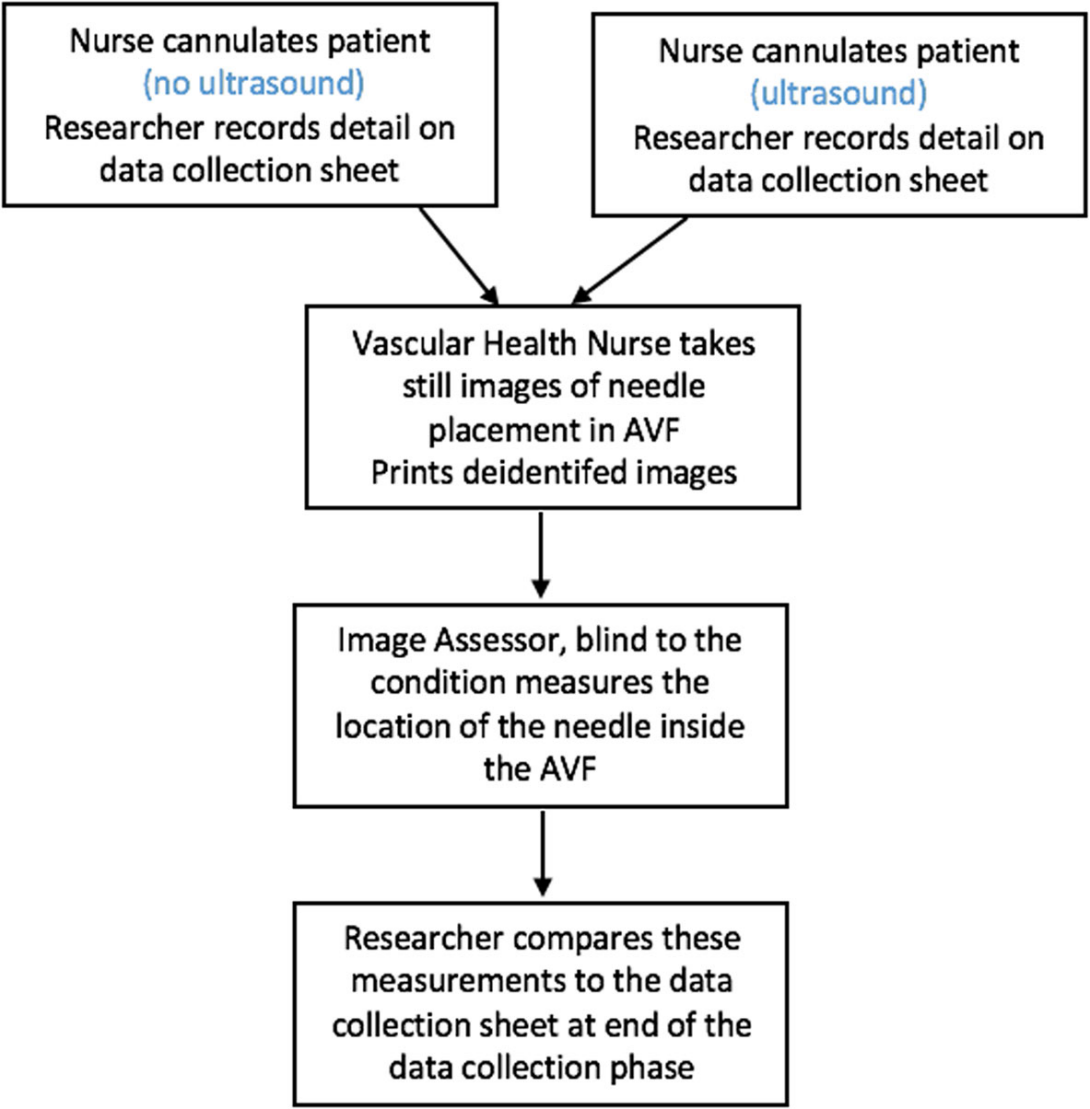
The procedure for assessment of needle placement

A third person (image assessor), who is experienced in renal vascular access, will review the images and record data on needle positioning. The image assessor will be blinded to patient, nurse, and condition and will measure (with a ruler) the location of the needle relative to the right and left side (transverse image only) of the vessel and the top and bottom of the vessel (transverse and longitudinal images). The image assessor will complete a short, structured survey at the end of the data collection phase to capture their perceptions about the ease of conducting measurements, the image quality, and their confidence in accuracy of their measurements (see Fig. 1).

Using Bowen et al.’s framework [15], Table 6 presents all the measures for which data are to be collected to inform the feasibility evaluation. The researcher will diarise events for the feasibility assessment, such as noting whether the protocol was adhered to by nurses (e.g. use of ultrasound) and patients (attendance and compliance with use of ultrasound). Feasibility assessment data will be collected via a structured survey at the completion of the data collection for all needle placement measurements. A semi-structured interview or focus group will be held following completion of the cannulation data collection phase to capture nurses’ perceptions of the feasibility of use of point-of-care ultrasound for routine cannulation. Questions will be asked in relation to ease of use, fit within the culture of the unit, barriers and facilitators, and suggestions for future implementation.

### Data analysis

Patients’ demographic and clinical data and nurses’ demographic and experience data will be reported as means and standard deviations or frequencies and proportions. The overall study feasibility, implementation feasibility, and acceptability will be reported using descriptive statistics. For feasibility, we will report the proportion of patients/nurses eligible for the study, the proportion of patients/nurses enrolled among those eligible, the reasons for declining participation, the proportion of patients/nurses completing the study, and the reasons for completing or not completing the study.

The implementation feasibility will be reported according to patients’ and nurses’ protocol adherence rate, the proportion of time that nurses adhered to the protocol, and the image assessor’s self-reported confidence regarding the accuracy of their measurements (Likert scale). Acceptability will be reported as the proportion of patients expressing satisfaction and the nurse satisfaction survey results related to the cannulation technique and use of point-of-care ultrasound-guided cannulation. Other data related to extravasations and nurse cannulation and ultrasound experience will be summarised using rates and proportions.

Data collected from semi-structured individual and focus groups interviews will be audio-recorded, transcribed verbatim, de-identified, and undergo content and thematic analyses based on a naturalistic inquiry approach.

The potential effect of the POCUS-guided cannulation on the clinical outcomes (miscannulations [0, 1, 2]), location of the needle in the vessel, and duration of the procedure will be estimated using generalised linear mixed models with link and distribution selected based on the type of outcome. The models will include the patient and nurse as random effects, and the condition, period (order in the sequence), and first carry-over effect as fixed effects.

## Discussion

Point-of-care ultrasound has been introduced into haemodialysis units sporadically throughout the world, but currently, there is no evidence to support or refute the use of POCUS guidance as an adjunct tool to use in the haemodialysis setting. This will be the first study of the use of POCUS guidance for cannulation in the haemodialysis setting. Testing the feasibility of this design is paramount to developing a comprehensive multi-site study in the future that could provide definitive outcomes in relation to the effectiveness of the use of POCUS guidance for cannulation in haemodialysis. Previous literature related to peripheral cannulation has provided some evidence of improved clinical outcomes related to the use of POCUS guidance for cannulation/insertion, but there were limitations in the designs of these studies, making it difficult to conduct meta-analyses.

Point-of-care ultrasound assessment and guided cannulation in haemodialysis have the potential to revolutionise the cannulation process. The vascular access is the patient’s lifeline; therefore, miscannulation and damage to the vessel lining associated with the standard practice of blind cannulation should not be accepted. Dialysis nurses have the responsibility to use tools that have the potential to promote best practice in cannulation of vascular access for haemodialysis. Benefits to patients may include, but are not limited to, avoidance of vessel damage, decreased thrombus and stenosis development, and avoidance of aborted dialysis sessions and insertion of CVDCs. Although this pilot and feasibility study will not provide outcomes related to effectiveness, it will provide a template to guide larger studies that will have the potential to produce definitive outcomes. If POCUS is found to be effective in ensuring correct needle placement and the multi-site study provides unequivocal evidence of the benefit of ultrasound-guided cannulation, then consideration should be given to the adoption of POCUS as standard practice in all haemodialysis settings.

## Abbreviations

ACTRN: Australian New Zealand Clinical Trials Registry
AVF: Arteriovenous fistula
AVG: Arteriovenous graft
CVAD: Central venous access device
POCUS: Point-of-care ultrasound
Qb: Dialysis machine pump speed
RCT: Randomised controlled trial
